# Alternative Measures of Body Composition and Outcomes Following Heart Transplant

**DOI:** 10.1111/ctr.70448

**Published:** 2026-01-30

**Authors:** Ana Lanier, Umar Siddiqi, Usmaan Siddiqi, Linda Lee, Seyed Ehsan Saffari, Mark Belkin, Jonathan Grinstein, Anthony Kanelidis, Leo Gozdecki, Stanley Swat, Sara Kalantari, Nitasha Sarswat, Bow Ben Chung, Gene Kim, Chris Salerno, Valluvan Jeevanandam, Manreet Kanwar, Ann Nguyen

**Affiliations:** ^1^ Section of Cardiology Department of Medicine University of Chicago Medicine Chicago USA; ^2^ Section of Cardiology Department of Medicine Northwestern University, Northwestern Medicine Chicago USA; ^3^ Department of Cardiac Surgery University of Chicago Medicine Chicago USA

**Keywords:** body composition, body mass index (BMI), heart transplant

## Abstract

Current guidelines for heart transplant (HT) listing support body mass index (BMI) < 35 kg/m^2^, though data supporting this recommendation is mixed. It is unclear if other more specific measurements of body composition are better predictors of outcomes post‐HT. This retrospective study included patients who underwent HT between 2014 and 2019 and underwent an abdominal CT scan within 3 months of HT. Tissue characterization was performed using Slice‐O‐Matic software to derive measurements including BMI, fat mass index (FMI), visceral adipose tissue to subcutaneous adipose tissue ratio (VAT/SAT), fat free mass index (FFMI), and skeletal muscle index (SMI). Univariate and multivariate logistic regression analyses were used to investigate association of body composition variables by tertiles to 1 year mortality, readmission, primary graft dysfunction, length of stay, infection, and renal failure. Of the 104 patients, 80% were male and 56% were Caucasian. Highest and lowest BMI tertiles were significantly associated with an increased risk of hospital readmission within 1 year post HT (OR 3.4, [95%CI 1.1–11.4]; OR 5.9, [95% CI 1.7–25.1]; *p* = 0.01) as well as an increased risk of infections requiring hospitalization (OR 3.2, [95% CI 1.2–9.0]; OR 3.9, [95%CI 1.4–11.7]; *p* = 0.02). FMI, VAT/SAT, FFMI, and SMI were not associated with outcomes 1‐year post‐HT. Highest and lowest BMI tertiles were associated with increased rates of hospital readmission and infection in the first‐year post HT, while other measurements of body composition were not associated with any poor outcomes post‐HT. Although BMI does not predict mortality post‐HT, it can differentiate risk better than all other metrics.

## 1 Introduction

Heart transplantation (HT) remains the gold standard treatment for patients with end‐stage heart failure. Current listing criteria guidelines support weight loss to achieve a body mass index (BMI) of ≤35 kg/m^2^ prior to listing, but outcomes data supporting this recommendation are mixed [1, 2, 3, 4, 5]. Moreover, BMI alone may fall short in identifying patients at higher risk for poor outcomes as it does not fully encapsulate body composition. With conflicting outcomes after HT demonstrated in obese patients, particularly in the BMI range just above transplant guidelines (BMI 35–40), questions remain whether more specific markers of body fat composition are better predictors of patient fitness and thus outcomes post HT [6].

Direct measurement of skeletal muscle mass and adipose tissue distribution may provide comprehensive information about the fitness of a patient. Conventional whole body composition profiling requires analysis at a three‐dimensional level using techniques such as computed tomography (CT), magnetic resonance imaging (MRI), and bone densitometry (DXA). CT and MRI, modalities frequently used in routine clinical care, are valuable tools for determining sarcopenia and body composition [7]. Many HT candidates undergo abdominal CT scans (abdCT) as part of their pre‐transplant workup to assess for other comorbidities [8]. This widespread availability of abdCT data provides a valuable resource for analyzing alternative measures of body composition without subjecting patients to additional imaging.

We sought to evaluate whether specific markers of body composition, which can be derived from direct segmentation of adipose tissue and skeletal muscle from abdCT scans, can serve as predictors of post‐transplant outcomes in HT patients. Our hypothesis was that other measurements beyond BMI could help identify patients at higher risk for poor outcomes after HT.

## 2 Materials and Methods

This single center, retrospective study was approved by the Institutional Review Board at University of Chicago Medical Center. All consecutive adult patients (≥18 years old) who underwent orthotopic HT from 2014‐2019 were screened. Retrospective chart review was performed, and the electronic health record was queried. Patients were included in the study if they had interpretable abdCT scans obtained within 3 months prior to HT. Patients were excluded from this study if abdCT scans were unavailable, acquired ≥3 months from transplant date, or were of poor image quality preventing adequate tissue characterization.

### 2.1 Tissue Characterization

Tissue characterization and fat and muscle segmentation were performed at the L3 level using Slice‐O‐Matic software v5.0 (TomoVision, Canada) [7]. Segmentation was performed using technique previously described by Mourtzakis et al. [9]. Utilizing this technique, skeletal muscle area (SMA, cm^2^), subcutaneous adipose tissue area (SAT, cm^2^), visceral adipose tissue area (VAT, cm^2^), and total fat area (TFA, cm^2^) were obtained at the L3 level. Derived equations were then used to calculate other measures of fat and muscle. Measurements of fat include whole body fat mass (WBFM, kg) calculated as ([0.042 × TFA] + 11.2), fat mass index (FMI, kg/m^2^) calculated as (WBFM/height), and VAT/SAT. Measurements of muscle include whole body fat free mass (WBFFM, kg) calculated as ([0.30 × TFA] + 6.06), fat free mass index (FFMI, kg/m^2^) calculated as (WBFFM/height), and skeletal muscle index (SMI, cm^2^/m^2^) calculated as (SMA/height). Values were reported by tertiles.

### 2.2 Clinical Characteristics

Pre‐transplant baseline demographic and clinical information was assessed. First year outcomes post‐transplant assessed included hospital readmission, rejection, primary graft dysfunction (PGD), index hospitalization length of stay (LOS), infection requiring hospitalization, renal failure requiring renal replacement therapy (RRT), post‐transplant de novo mechanical circulatory support (MCS), and death from any cause.

Baseline characteristics and clinical outcomes were reported as mean ± standard deviation or median with minimum and maximum. Comparisons between continuous variables were performed using a two‐sample *t*‐test or the Mann–Whitney *U* test, depending on the tenability of the normality assumption. Categorical variables were presented as frequency and percentages and compared between the above two groups using Chi‐square or Fisher's exact test, where appropriate. Univariate logistic regression analysis was performed to investigate the association of baseline body composition variables (treated as tertiles) and clinical outcomes. Multivariate logistic regression analysis was conducted, where appropriate, to adjust for potential clinical confounders.

Unadjusted and adjusted odds ratios, 95% confidence intervals, and *p* values were reported. Goodness‐of‐fit of the models was assessed via Hosmer–Lemeshow statistics. Statistical significance was set at *p* < 0.05. Descriptive statistics and multivariate logistic regression were performed using R statistical software (v4.1.1).

## 3 Results

### 3.1 Baseline Characteristics

A total of 104 patients were included in this study: 80% male, 56% Caucasian with median age at transplant of 57 (18–72) years. Most patients had a non‐ischemic cardiomyopathy (69.2%) and received basiliximab induction (67.3%). A total of 35 (34%) patients underwent multiorgan transplant; 19 (18%) heart‐kidney, 9 (8%) heart‐liver, and 7 (6%) heart‐liver‐kidney. Prior to HT, 81.2% of patients were supported with a mechanical device, and 50% were supported with one or more inotropes. Baseline characteristics are shown in Table 1.

**TABLE 1:** Baseline characteristics.

Baseline characteristics	*N* = 104
Age at transplant, years	57 (18–72)
Gender, male	83 (79%)
Height (cm)	175 (145–198)
Weight (kg)	83.5 (48.9–129)
BMI (kg/m^2^)	27.8 (17.5–42.7)
Tertile 1, range	17.5–24.3
Tertile 2, range	24.4–30.2
Tertile 3, range	30.2–42.7
Race	
White	57 (54%)
African American	31 (29%)
Multiorgan transplant	35 (33%)
Heart‐Kidney	19 (18%)
Heart‐Liver	9 (8%)
Heart‐Liver‐Kidney	7 (6%)
Heart failure etiology	
Ischemic cardiomyopathy	32 (30%)
Nonischemic cardiomyopathy	70 (60%)
Comorbidities	
Coronary artery disease	49 (47%)
Atrial fibrillation	55 (53%)
Hypertension	52 (50%)
Diabetes	35 (33%)
Chronic kidney disease	55 (52%)
Cirrhosis	15 (14%)
Stroke	8 (7.7%)
Pre‐transplant MCS	
IABP/Impella	68 (65%)
Durable LVAD	16 (15%)
ECMO	4 (3.8%)
1+ inotropes	53 (51%)
Induction	
None	25 (24%)
Basiliximab	70 (67.3%
Thymoglobulin	9 (8.7%)
Donor ischemic time (min)	244 (120–405)

*Note:* Characteristics reported as median (range), mean ± standard deviation, or total (percentage) as appropriate.

Abbreviations: ECMO, extracorporeal membrane oxygenation; IABP, intraaortic balloon pump; LVAD, left ventricular assist device; MCS, mechanical circulatory support.

### 3.2 Clinical Outcomes

#### 3.2.1 BMI

**FIGURE 1:**
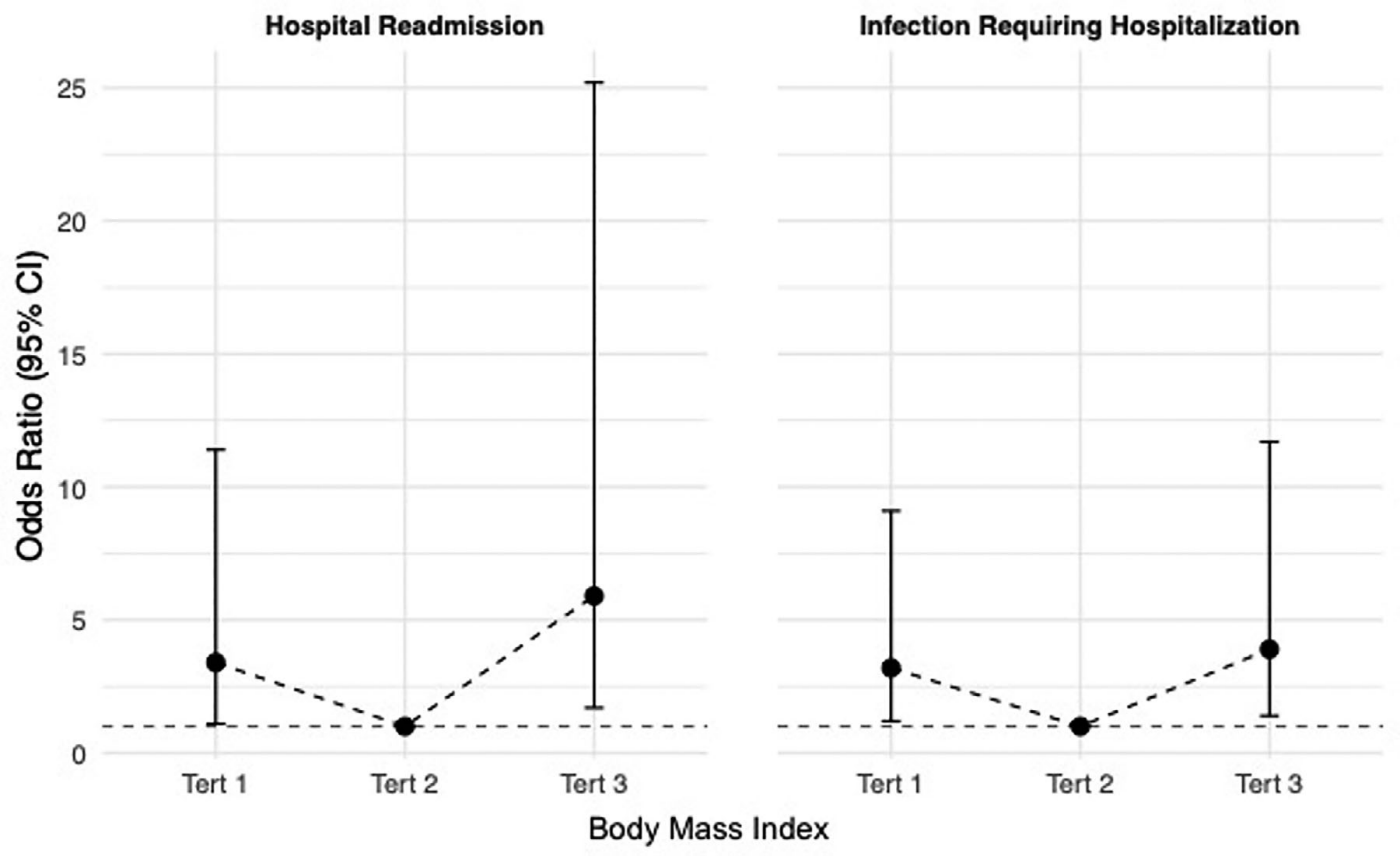
Association of body mass index (BMI) with hospital readmission and infections requiring hospitalization. CI (confidence interval); Tert‐1, tertile 1; Tert‐2, tertile 2; Tert‐3, tertile 3. Values are reported as odds ratio with 95% confidence interval and are relative to Tertile 2 in each respective outcome figure. All reported values are significant, defined as *p* < 0.05.

The median BMI was 27.8 kg/m^2^ (17.5–42.7 kg/m^2^). BMI was significantly associated with hospital readmission following HT, with both higher and lower BMI tertiles showing increased risk of hospital readmission (Tert‐1 OR 3.4 [95% CI, 1.1–11.4]; Tert‐3 OR 5.9 [95% CI, 1.7–25.2]; *p* = 0.01). BMI was also significantly associated with an increased risk of infections requiring hospitalization, with both higher and lower BMI tertiles showing increased risk (Tert‐1 OR 3.2 [95% CI, 1.2–9.1]; Tert‐3 OR 3.9 [95% CI 1.4–11.7]; *p* = 0.02) (Figure 1). However, tertiles of BMI were not significantly associated with other post‐transplant outcomes at 1‐year including rejection, PGD, index hospitalization LOS, renal failure requiring RRT, post‐transplant de novo MCS, or death.

#### 3.2.2 Measures of Fat

Median values of fat measurements include FMI 9.15 kg/m^2^ (3.53–15.7 kg/m^2^), and VAT/SAT 0.64 (0.06–6.87). FMI and VAT/SAT were not significantly associated with any post‐transplant outcomes including hospital readmission, rejection, PGD, index hospitalization LOS, infections requiring hospitalization, renal failure requiring RRT, post‐transplant de novo MCS, and death at 1 year.

#### 3.2.3 Measures of Muscle

Median values of measurements of muscle include FFMI 16.89 kg/m^2^ (1.91–29.23 kg/m^2^), and SMI 49.64 cm^2^/m^2^ (33.48–88.99 kg/m^2^). FFMI and SMI were not significantly associated with any post‐transplant outcomes including hospital readmission, rejection, PGD, index hospitalization LOS, infections requiring hospitalization, renal failure requiring RRT, post‐transplant de novo MCS, and death at 1 year.

## 4 Discussion

Given inconsistent data regarding BMI on mortality after HT, this study attempted to identify if other markers of body composition would be better predictors of several key post‐HT outcomes. We found that BMI was significantly associated with both hospital readmission and infection requiring hospitalization with both the highest and lowest BMI tertiles showing increased risk of hospital readmission and infection requiring hospitalization following transplant. Other body composition variables were not significantly associated with poor outcomes at 1‐year post‐HT defined as increased risk of hospitalization, infection requiring hospital admission, PGD, index hospitalization LOS, renal failure requiring RRT, or death.

Alternative measures in body composition have been utilized across multiple medical disciplines including solid organ transplant, oncology, and preventative healthcare. Both CT and DXA have been accepted as imaging modalities to quantify skeletal muscle mass and adipose tissue [7, 9, 10]. In contrast to CT, DXA is less expensive, with lower radiation exposure, but cannot differentiate between VAT and SAT [10]. As an alternative, methods of assessing abdominal adiposity via a single sliced image at the L4‐L5 intervertebral space obtained from abdCT have been developed to reduce cost and radiation dose [10, 11]. Both skeletal muscle mass and SAT, as well as derived calculated ratios of body composition, have been established to assess outcomes across a variety of disciplines. Historically, these body composition metrics such as FMI, WBFM, and BMI were predominantly utilized in assessing oncologic outcomes [12, 13].

There has been a push away from BMI as the sole measure of clinical and surgical risk after transplantation, with growing research suggesting alternative measures provide more specific risk‐stratification [14, 15, 16]. This includes lower SMI being shown to be a marker of increased morbidity and mortality in older men undergoing renal transplantation as well as higher VAT being associated with risk of early allograft dysfunction in liver transplantation [17, 18]. Additionally, low muscle mass has been shown to strongly correlate with mortality after liver transplant. These findings were consistent across multiple modalities used to derive body composition measurements, including CT‐derived psoas muscle area and bioelectrical impedance analysis [15, 19]. Research on alternative measures of body composition specifically in the context of HT mortality has been more limited. One study did show that DEXA‐derived WBFM and FMI were associated with post‐operative maximal exercise capacity after HT [20]. We evaluated multiple measurements of fat and muscle on HT outcomes and did not find any association.

Guidelines for HT listing are currently based on BMI. Previously, the International Society for Heart and Lung Transplantation guidelines for HT listing recommended a goal BMI < 30 kg/m^2^ [21]. This was based on data showing morbid obesity as an independent predictor of mortality in post‐HT [14, 15, 22]. These guidelines were updated in 2016 to recommend BMI <35 kg/m^2^ after studies showed no significant difference in morbidity and mortality following HT in those with BMI 30–34.99 kg/m^2^ [1, 3]. Instead, a multimodal approach is suggested for pre‐operative optimization, noting recent UNOS data suggesting the relationship between BMI and post‐transplant mortality was u‐shaped with increased risk in patients with both low BMI (<18.5 kg/m^2^) and high BMI (>35 kg/m^2^) [1, 3, 23]. This BMI threshold continues to be unclear as an even more recent study shows acceptable outcomes in the BMI range of 35–39.9 kg/m^2^, demonstrating that BMI overall may not be a reliable parameter for predicting mortality [6]. This lack of specificity becomes even more relevant in a patient population predisposed to weight and body composition fluctuations in the setting of volume overload and heart failure driven cachexia. Our study adds to the body of data that shows that BMI does not predict mortality after HT.

BMI was found in this study to be associated with infection and readmission following HT in a u‐shaped pattern. To date, there has been no published research demonstrating these findings in HT, though similar findings have been seen in liver and lung transplants with increased risk of infection demonstrated in both overweight and obese patients as well as low weight patients [24, 25]. Research evaluating the association of BMI with increased admissions has been less revealing though notably, markers associated with low weights such as low muscle mass and frailty markers have been shown to have increased rates of hospital readmission following kidney transplant [26, 27]. Obesity has long been associated with increased infection risk, with proposed physiology including higher rates of comorbidities, with risk factors including chronic inflammation and immune dysregulation [28, 29]. Similar trends have been seen in low BMI patients, with the primary proposed driver being malnutrition [30]. These effects were demonstrated independent of immune status, so theoretically would be more pronounced with use of immunosuppressive medication. Although reason for hospitalization was not captured in this study, it is possible the increased admission rate was driven by infection (Figure 1).

In addition to the retrospective, single‐center nature of this study, the main limitation of this analysis is the sample size. This limited the ability to stratify by sex which may have prevented capturing sex‐specific differences in body composition while also limiting the number of confounding variables adjusted for in the multivariate analysis. We did, however, adjust for sex in the multivariate analysis. Finally, this does not consider other factors known to impact post‐HT outcomes that can be associated with obesity or low muscle mass such as insulin resistance or malnutrition.

## 5 Conclusion

BMI was significantly associated with increased risk of infection and readmission in the year following HT. Alternative measures of body composition outside of BMI were not significantly associated with clinical outcomes post HT. Furthermore, BMI was not associated with mortality and therefore may be better served as a risk assessment tool rather than a definitive guide for listing. Given growing research supporting the expansion of our definition of body composition beyond BMI, continued work to understand the impact of body composition on HT outcomes is necessary and could provide targeted approaches to pre‐transplant optimization.

## Conflicts of Interest

The authors declare no conflicts of interest.

## Supporting information




**Supplemental Figure 1**. One‐Year Readmissions. Table includes all‐cause readmissions as well as sub‐analysis of readmissions due to infection.

**Supplemental Table 1**: Outcomes by Body Composition. Abbreviations: BMI = Body Mass Index; VAT (visceral adipose tissue), SAT (Subcutaneous adipose tissue), FMI (fat mass index), FFMI (fat free mass index), PGD (Primary Graft Dysfunction), LOS (Length of Stay); OR = Odds Ratio; CI = Confidence Interval; NE = Not Estimable. Significance defined as P < 0.05, denoted with **.

**Supplemental Table 2**: Sub‐analysis of data using the entire cohort (n = 104) adjusting for multiorgan transplant status as a covariate in all regression models. Abbreviations: BMI = Body Mass Index; VAT (visceral adipose tissue), SAT (Subcutaneous adipose tissue), FMI (fat mass index), FFMI (fat free mass index); OR = Odds Ratio; CI = Confidence Interval; NE = Not Estimable. Significance defined as P < 0.05, denoted with **.

## Data Availability

The data that support the findings of this study are available from the corresponding author upon reasonable request.
