# Supplementary material for: Alternative Measures of Body Composition and Outcomes Following Heart Transplant
Source: Clin Transplant. 2026 Jan 30;40(2):e70448. doi: 10.1111/ctr.70448 (PMC12857596; doi:10.1111/ctr.70448)
Supplement: Supplementary file 1 — Supplemental Figure 1. One‐Year Readmissions. Table includes all‐cause readmissions as well as sub‐analysis of readmissions due to infection. [file CTR-40-e70448-s001.docx]

**Supplemental Figure 1 :** One-Year Readmissions. Table includes all-cause readmissions as well as sub-analysis of readmissions due to infection.
