# Supplementary material for: Alternative Measures of Body Composition and Outcomes Following Heart Transplant
Source: Clin Transplant. 2026 Jan 30;40(2):e70448. doi: 10.1111/ctr.70448 (PMC12857596; doi:10.1111/ctr.70448)
Supplement: Supplementary file 2 — Supplemental Table 1: Outcomes by Body Composition. Abbreviations: BMI = Body Mass Index; VAT (visceral adipose tissue), SAT (Subcutaneous adipose tissue), FMI (fat mass index), FFMI (fat free mass index), PGD (Primary Graft Dysfunction), LOS (Length of Stay); OR = Odds Ratio; CI = Confidence Interval; NE = Not Estimable. Significance defined as P < 0.05, denoted with **. [file CTR-40-e70448-s003.docx]

| Characteristics | | Infection Requiring  Hospitalization | | Renal Failure  Requiring RRT | | Post-Transplant  De Novo MCS | | Hospital  Readmission | | Rejection | | | Death  at One Year | | PGD | | | | Index Hospitalization LOS | | |  |
| --- | --- | --- | --- | --- | --- | --- | --- | --- | --- | --- | --- | --- | --- | --- | --- | --- | --- | --- | --- | --- | --- | --- |
|  |  | OR (95% CI) | P value | OR (95% CI) | P value | OR (95% CI) | P value | OR (95% CI) | P value | OR (95% CI) | P value | OR (95% CI) | | P value | | OR (95% CI) | P Value | OR (95% CI) | | P Valus |  |  |
| BMI | *Tert 1 vs Tert 2* | 3.2 (1.2 - 9.1) | **0.02**** | 0.9 (0.2 - 5.6) | >0.9 | 0.9 (0.3 - 2.5) | 0.90 | 3.4 (1.1 - 11.4) | **0.01**** | 0.8 (0.3 - 2.4) | 0.94 | 0.3 (0.01 - 2.5) | | 0.45 | | 1.8 (0.6-5.9) | 0.34 | 0.9 (0.3-2.3) | | 0.55 |  |  |
|  | *Tert 3 vs Tert 2* | 3.9 (1.4 - 11.7) |  | 2.6 (0.7 - 12.9) | 0.2 | 0.8 (0.3 - 2.3) |  | 5.9 (1.7 - 25.2) |  | 0.9 (0.3 - 2.8) |  | 0.3 (0.01-2.51) | |  |  | 0.8 (0.2-2.9) |  | 1.5 (0.6-3.9) | |  |  |  |
|  |  |  |  |  |  |  |  |  |  |  |  |  | |  | |  |  |  | |  |  |  |
| FMI | *Tert 1 vs Tert 2* | 1.2 (0.4 - 3.2) | 0.95 | 0.7 (0.13 - 3.4) | 0.7 | 0.8 (0.3 - 2.3) | 0.46 | 0.9 (0.3 - 3.03) | 0.99 | 0.8 (0.3 - 2.4) | 0.93 | 2 (0.2 - 44.3) | | 0.83 | | 1.8 (0.6-5.9) | 0.34 | 0.6 (0.2-1.7) | | 0.31 |  |  |
|  | *Tert 3 vs Tert 2* | 1.1 (0.4 - 2.9) |  | 1.6 (0.4 - 6.6) | 0.5 | 0.5 (0.2 - 1.5) |  | 0.9 (0.3 - 3.1) |  | 0.9 (0.3 - 2.9) |  | 2 (0.2 - 44.3) | |  |  | 0.8 (0.2-2.9) |  | 1.3 (0.5-3.5) | |  |  |  |
|  |  |  |  |  |  |  |  |  |  |  |  |  | |  | |  |  |  | |  |  |  |
| VAT/SAT | *Tert 1 vs Tert 2* | 1.2 (0.4 - 3.3) | 0.91 | 0.4 (0.1 - 1.4) | 0.2 | 0.9 (0.3 - 2.7) | 0.94 | 2.3 (0.7 - 7.9) | 0.29 | 2.2 (0.7 - 8.2) | 0.21 | 0.3 (0.01 - 2.5) | | .45 | | 6.9 {1.6-47.3) | 0.06 | 1.3 (0.5-3.4) | | 0.38 |  |  |
|  | *Tert 3 vs Tert 2* | 1.2 (0.5 - 3.5) |  | 0.4 (0.1 - 1.4) | 0.2 | 1.1 (0.4 - 3.2) |  | 1.9 (0.6 - 6.5) |  | 3.0 (0.9 - 11.4) |  | 0.3 (0.01 - 2.5) | |  |  | 4.4 (1-31.2) |  | 0.7 (0.2-1.8) | |  |  |  |
|  |  |  |  |  |  |  |  |  |  |  |  |  | |  | |  |  |  | |  |  |  |
| FFMI | *Tert 1 vs Tert 2* | 1.1 (0.4 - 2.9) | 0.94 | 1.7 (0.4 - 9.0) | 0.5 | 0.9 (0.3 - 2.7) | 0.93 | 1.2 (0.4 - 3.6) | 0.72 | 1.1 (0.3 - 3.2) | 0.96 | NE | | >0.9 | | 0.5 (0.1-1.6) | 0.52 | 1.3 (0.5-3.5) | | 0.48 |  |  |
|  | *Tert 3 vs Tert 2* | 0.9 (0.3 - 2.4) |  | 1.7 (0.4 - 9.0) | 0.5 | 0.8 (0.3 - 2.4) |  | 1.6 (0.5 - 5.7) |  | 1.2 (0.4 - 3.5) |  | NE | |  |  | 0.7 (0.2-2.1) |  | 0.7 (0.3-1.9) | |  |  |  |
|  |  |  |  |  |  |  |  |  |  |  |  |  | |  | |  |  |  | |  |  |  |
| SMI | *Tert 1 vs Tert 2* | 0.9 (0.4 - 2.6) | 0.74 | 2.7 (0.5 - 19.6) | 0.3 | 1.5 (0.5 - 4.4) | 0.79 | 1.1 (0.4 - 3.5) | 0.74 | 1.2 (0.4 - 3.8) | 0.69 | NE | | >0.9 | | 0.5 (0.1-1.6) | 0.52 | 1.3 (0.5-3.5) | | 0.52 |  |  |
|  | *Tert 3 vs Tert 2* | 0.7 (0.3 - 1.8) |  | 3.3 (0.7 - 23.8) | 0.2 | 1.3 (0.4 - 4.0) |  | 1.6 (0.5 – 5.4) |  | 1.6 (0.6 - 4.9) |  | NE | |  |  | 0.6 (0.2-2.1) |  | 0.8 (0.3-2) | |  |  |  |
| **Supplemental Table 1**: Outcomes by Body Composition. Abbreviations: BMI=Body Mass Index; VAT (visceral adipose tissue), SAT (Subcutaneous adipose tissue), FMI (fat mass index), FFMI (fat free mass index), PGD (Primary Graft Dysfunction), LOS (Length of Stay); OR=Odds Ratio; CI=Confidence Interval; NE=Not Estimable. Significance defined as P<0.05, denoted with **. | | | | | | | | | | | | | | | | | | | | | | |
