# Supplementary material for: Alternative Measures of Body Composition and Outcomes Following Heart Transplant
Source: Clin Transplant. 2026 Jan 30;40(2):e70448. doi: 10.1111/ctr.70448 (PMC12857596; doi:10.1111/ctr.70448)
Supplement: Supplementary file 3 — Supplemental Table 2: Sub‐analysis of data using the entire cohort (n = 104) adjusting for multiorgan transplant status as a covariate in all regression models. Abbreviations: BMI = Body Mass Index; VAT (visceral adipose tissue), SAT (Subcutaneous adipose tissue), FMI (fat mass index), FFMI (fat free mass index); OR = Odds Ratio; CI = Confidence Interval; NE = Not Estimable. Significance defined as P < 0.05, denoted with **. [file CTR-40-e70448-s002.docx]

| Characteristics | | Infection Requiring  Hospitalization | | Renal Failure  Requiring RRT | | Post-Transplant  De Novo MCS | | Hospital  Readmission | | Rejection | | | Death  at 1 Year | | |  |
| --- | --- | --- | --- | --- | --- | --- | --- | --- | --- | --- | --- | --- | --- | --- | --- | --- |
|  |  | OR (95% CI) | P value | OR (95% CI) | P value | OR (95% CI) | P value | OR (95% CI) | P value | OR (95% CI) | P value | OR (95% CI) | | P value |  |  |
| BMI | *Tert 1 vs Tert 2* | 3.2 (1.2, 9.1) | **0.021** | 0.8 (0.1, 4.9) | 0.27 | 0.9 (0.3, 2.5) | 0.9 | 4.9 (1.5, 18.8) | **<0.01** | 1.1 (0.3, 3.4) | >0.99 | 0.2 (0.0, 1.8) | | 0.29 |  |  |
|  | *Tert 3 vs Tert 2* | 3.9 (1.4, 11.7) |  | 2.4 (0.6, 12.3) |  | 0.8 (0.3, 2.3) |  | 7.9 (2.1, 37.2) |  | 1.1 (0.3, 3.4) |  | 0.2 (0.0, 2.0) | |  |  |  |
|  |  |  |  |  |  |  |  |  |  |  |  |  | |  |  |  |
| FMI | *Tert 1 vs Tert 2* | 1.2 (0.4, 3.2) | 0.95 | 0.5 (0.1, 2.6) | 0.4 | 0.8 (0.3, 2.3) | 0.46 | 1.2 (0.4, 4.1) | 0.94 | 1.4 (0.4, 4.6) | 0.85 | 1.2 (0.1, 27.3) | | 0.87 |  |  |
|  | *Tert 3 vs Tert 2* | 1.1 (0.4, 2.9) |  | 1.5 (0.4, 6.4) |  | 0.5 (0.2, 1.5) |  | 1 (0.3, 3.3) |  | 1.1 (0.4, 3.5) |  | 1.8 (0.2, 41.6) | |  |  |  |
|  |  |  |  |  |  |  |  |  |  |  |  |  | |  |  |  |
| VAT/SAT | *Tert 1 vs Tert 2* | 1.2 (0.4, 3.3) | 0.91 | 0.4 (0.1, 1.6) | 0.34 | 0.9 (0.3, 2.7) | 0.94 | 2.2 (0.7, 7.7) | 0.37 | 2 (0.6, 7.9) | 0.33 | 0.4 (0.0, 3.3) | | 0.63 |  |  |
|  | *Tert 3 vs Tert 2* | 1.2 (0.4, 3.5) |  | 0.4 (0.1, 1.7) |  | 1.1 (0.4, 3.2) |  | 1.8 (0.6, 5.9) |  | 2.6 (0.8, 10.4) |  | 0.4 (0.0, 3.8) | |  |  |  |
|  |  |  |  |  |  |  |  |  |  |  |  |  | |  |  |  |
| FFMI | *Tert 1 vs Tert 2* | 1.1 (0.4, 2.9) | 0.94 | 1.7 (0.4, 9.1) | 0.75 | 0.9 (0.3, 2.7) | 0.93 | 1.1 (0.4, 3.6) | 0.68 | 1 (0.3, 3.2) | 0.92 | NE | | >0.99 |  |  |
|  | *Tert 3 vs Tert 2* | 0.9 (0.3, 2.4) |  | 1.7 (0.4, 9.1) |  | 0.8 (0.3, 2.4) |  | 1.7 (0.5, 6.1) |  | 1.2 (0.4, 3.9) |  | NE | |  |  |  |
|  |  |  |  |  |  |  |  |  |  |  |  |  | |  |  |  |
| SMI | *Tert 1 vs Tert 2* | 1 (0.4, 2.6) | 0.74 | 2.6 (0.5, 19.3) | 0.36 | 1.5 (0.5, 4.4) | 0.79 | 1.1 (0.4, 3.6) | 0.74 | 1.2 (0.4, 4.0) | 0.71 | NE | | >0.99 |  |  |
|  | *Tert 3 vs Tert 2* | 0.7 (0.3, 1.9) |  | 3.4 (0.7, 25.2) |  | 1.3 (0.4, 4.0) |  | 1.6 (0.5, 5.6) |  | 1.6 (0.5, 5.4) |  | NE | |  |  |  |
|  | | | | | | | | | | | | | | | | |

**Supplemental Table 2:** Sub-analysis of data using the entire cohort (n=104) adjusting for multiorgan transplant status as a covariate in all regression models. Abbreviations: BMI=Body Mass Index; VAT (visceral adipose tissue), SAT (Subcutaneous adipose tissue), FMI (fat mass index), FFMI (fat free mass index); OR=Odds Ratio; CI=Confidence Interval; NE=Not Estimable*.* Significance defined as P<0.05, denoted with **.
